# Assessment of Carcinogenic and Non-Carcinogenic Risk Lead in Bottled Water in Different Age Groups in Bandar Abbas Ciry, Iran

**DOI:** 10.5539/gjhs.v7n4p286

**Published:** 2015-01-23

**Authors:** Yadolah Fakhri, Seyed Mohsen Mohseni, Saeedeh Jafarzadeh, Ghazaleh Langarizadeh, Bigard Moradi, Yahya Zandsalimi, Aziz Rahimizadeh, Maryam Mirzaei

**Affiliations:** 1 Social Determinants in Health Promotion Research Center, Hormozgan University of Medical Sciences, Bandar Abbas, Iran; 2 Department of Environmental Health Engineering, School of Public Health, Qom University of Medical Sciences, Qom, Iran; 3 Research Center for Non-Communicable Disease, Fasa University of Medical Sciences, Fasa, Iran; 4 Food and Drugs Research Center, Bam University of Medical Sciences, Bam, Iran; 5 Department of Health Public, Kermanshah University of Medical Sciences, Kermanshah, Iran; 6 Environmental Health Research Center, Kurdistan University of Medical Science, Sanandaj, Iran; 7 Jahrom University of medical sciences, Jahrom, Iran

**Keywords:** lead, bottled waters, carcinogenic, non-carcinogenic, drinking water

## Abstract

The presence of heavy metals such as lead in drinking water resources can be dangerous for human because of toxicity and biological accumulation. The consumption of water or food which contains lead in high concentration can lead to prevent from Hemoglobin Synthesis (Anemia) and Kidney diseases. In this present study, the researcher collected 432 samples of bottled water in the popular marks in summer and winter from the surface of Bandar Abbas. The lead concentration was measured by atomic absorption Spectrophotometer in model DR2800 through the Dithizone method. CDI, R and HQ which are caused by lead for adult men, women and children, have been calculated and evaluated through the equations of EPA and WHO. The mean concentration of lead, which is 3.46±0.47 µg/l, and its range, which is 1.9-17.6 µg/l, are lower than the guideline of WHO (10 µg/l) and MPC of EPA is (15 µg/l). But the 40 samples of the bottled water (9.2%) have the concentration higher than guideline WHO and 8 samples (1.85%) has the concentration higher than the permissible limits of the EPA. CDI in different age groups is as following manner: Children>adult men>adult women. CDI in children is more than twice as much as in the adult men and women. The R of lead for children (24E-7), adult men (11E-7) and for adult women (10E-7) are more than the acceptable level of R in EPA (1E-6) but less than the acceptable level of R in WHO (1E-4). Since HQ of adult men (34E-5), adult women (31E-5) and children (84E-5), is lower than 1, it can be said that the population of Bandar Abbas is in a safe area regarding the HQ of the bottled water’s lead.

## 1. Introduction

The heavy metals are the elements with a special weight 4-5 times as much as water (Duruibe, Ogwuegbu, & Egwurugwu, 2007; Raikwar, Kumar, M. Singh, & A. Singh, 2008). These elements have biological accumulation, toxicity, and environmental sustainability properties (Pekey, Karakaş, & Bakoglu, 2004). In recent years, the presence of these metals such as Arsenic (As), Cadmium (Cd), Mercury (Hg), lead (Pb), Nickel (Ni), and Chrome (Cr) in drinking water have become an international environmental and health concern (Dogaru, Zobrist, Balteanu, Popescu, & Sima, 2009; Z. Wang, Chai, Y. Wang, Yang, & H. Wang, 2011; Ghaderpouri, Jahed Khaniki, & Nazmara, 2009). The entry of the heavy metals in water resources can be due to the natural processes such as wastewater municipal, industrial, and agricultural sewage (Demirak, Yilmaz, Levent Tuna, & Ozdemir, 2006). The heavy metals naturally exist in small amount in water. Many of these elements have a dual role in the human body (Fiket, Roje, Mikac, & Kniewald, 2007). The heavy metals could be dangerous for human health at higher values than the standard (Ahmad, Mushrifah, & Othman, 2009; Khan, Ahmad, & Rahman, 2007). The epidemiological studies show that there is a significant relationship between tooth decay, kidney disorders, neurological disorders, and cancers with heavy metals (Soupioni, Symeopoulos, & Papaefthymiou, 2006; Babaji, Shashikiran, & Reddy, 2004). The lead poisoning can prevent proper functioning of the kidneys, reproductive system, circulatory system, acute and chronic damage to the central and peripheral nervous system (Duruibe, Ogwuegbu, & Egwurugwu, 2007; Singh, Sharma, Agrawal, & Marshall, 2010; Järup, 2003). In the past thirty years, the packaged water consumption has been rising in many communities (Karamanis, Stamoulis and Ioannides, 2007; Jakus, Shaw, Nguyen, & Walker, 2009). The packaged drinking water is divided into either mineral or bottled water. In the bottled water, various additives such as sodium, calcium, carbonates, and etc is useful for body humans. But in the mineral water, the water bottles are filled with water fountain without any additives (Cybulska & Doe, 2006).

Many studies have measured the heavy metals concentration in drinking water and compared it with standard value (Arab & Alshikh, 2010; Shotyk & Krachler, 2007; Dabeka, Conacher, Lawrence, Newsome, & McKenzie, 2002; Ikem, Odueyungbo, Egiebor, & Nyavor, 2002). Also, in some studies, carcinogenic (R) and non-carcinogenic risk (HQ) of the heavy metals which are due to drinking water consumption, have been assessed (Wang at al., 2011; Jakus, Shaw, Nguyen, & Walker, 2009; Muhammad, Shah, & Khan, 2011). This study was an attempt to measure the lead concentration in popular marks of bottled water and it makes comparison with the standard value because of sanitary importance of lead in drinking water and also the excessive consumption of water, especially the bottles water in Bandar Abbas and after it, the, R and HQ in the age groups of adult men, adult women and children calculated and assessed.

## 2. Method

### 2.1 Area Study

The coastal city of Bandar Abbas (Center of Hormozgan Province) is located at the south of Iran (54°22´7” and 27°11´53”) and at a height of 9 meters above the sea level (Darvishsefat & Tajvidi, 2006). The climate of this city is hot and humid and its population is growing by the day because of economic and industrial development.

### 2.2 Sample Collection

This descriptive sectional study was conducted in summer and winter 2013. The sample collection was made from 8 marks of popular packaged water in Bandar Abbas at 13 different places. Per month 9 number of 1.5 liter was selected randomly from each mark. 216 samples of water were collected in summer and 216 samples were collected in winter (total 432 samples of bottled water). The samples were transferred to the chemistry laboratory of health faculty of Medial Science, University of Hormozgan in 4-6 °C in order to measure the heavy metals concentration (Eaton, Clesceri, & Greenberg, 2005).

**Figure 1:**
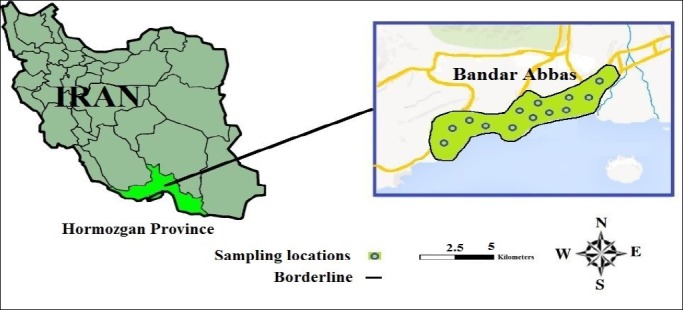
Areas of collecting samples of bottled water in the surface of Bandar Abbas in the south of Iran

### 2.3 Measurement Concentration of Lead

1 ml nitric acid was added to water samples per each liter of sample water in a laboratory to get to PH<2 (to save up the lead up to 28 days in the water samples). For condensation, water samples were passed through whatsman glass micro-fiber filter (Muhammad, Shah, & Khan, 2011). The lead concentration was measured by atomic absorption Spectrophotometer in model DR2800 through the Dithizone method (Hach, 2007).

### 2.4 Statistical Analysis

The difference of mean concentration of lead in different marks of the bottled water, the difference of lead concentration in summer and winter, the difference of R and HQ in different age groups was analyzed by T- test and one-way ANOVA test by software SPSS16. An error of 5% (α=5) was considered as significant level.

### 2.5 Risk Assessment

The calculation of the Chronic Daily Intake (CDI) was performed through the equation which was presented by the United States Environmental Protection Agency (USEPA): (Forum, 2005; Kavcar, Sofuoglu, & Sofuoglu, 2009).


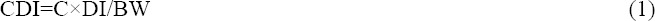


In this equation, CDI is Chronic Daily Intake (mg/kg-d), C is the concentration of lead in drinking water (µg/l), DI is mean daily intake water (l/d), and BW is a body weight (kg). Because of, not exist data about DI and BW of Population in Bandar Abbas; we used date suggested by WHO and EPA. Hence, DI for adult men (17-65 years old), adult women (17-65 years old) and children (4-14 years old) 2.723, 2.129, 1.8 l/d and BW 76, 64 and 22.3 kg, are respectively (EPA, 2004; WHO, 2006). The R of the heavy metals due to eating or drinking was calculated by Equation 2 (Patrick, 1994).


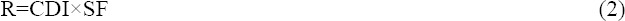


In this equation, R is non-carcinogenic risk during lifetime, resulting from exposure to contaminants (Pb), CDI is the Chronic Daily Intake (mg/kg-d), and SF is the safety factor of contaminants slope (for lead is 0.0085 kg-d/mg). The acceptable level of R according to EPA and WHO is respectively less than 1E-6 (One cancer in 1000000 people) and less than 1E-4 (One cancer in 10000 people) (EPA, 2006; WHO, 2004). Also, the Hazard Quotient (HQ) was computed for the calculation of the HQ of lead in drinking water by the Equation 3:


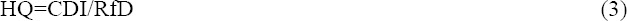


In Equation 3, RFD is dose of polluter’s reference (mg/kg-d) which was considered 0.36 mg/kg-d for lead (Environmental Protection Agency, April 1992; Revised in December 2012). A population is located in a safe area when Hazard Quotient is less than 1: HQ<1 (Hashmi, Yu, Shen, Duan, & Shen, 2013). 

## 3. Result

The mean and range of lead concentration in the samples of the bottled drinking water is 3.46±0.47 µg/l and 1.9-17.6 µg/l, respectively (432 samples). The mean concentration of lead in marks BW1, BW2, BW3, BW4, BW5, BW6, BW7, and BW8 were 7.33± 88, ND, 2.5±0.57, 0.5± 0.06, 4.83±0.68, 5.5±0.89, 2.33±0.34 and 4.68 ± 0.51, respectively (See Table 1). The range of concentration in mark BW1 was observed higher than the standard of EPA and WHO and the mark BW6 is higher than WHO standard (See Figure 1). According to the guideline of WHO (10 µg/l) and MPC^1^ of EPA (15 µg/l), the mean concentration of lead in all the marks is less than the standards.

**Table 1:** Mean (M±SE), Standard deviation (SD), the range of lead concentration (µg/l), in 8 marks of the bottled water in Bandar Abbas in summer and winter 2013 (n=432)

	Summer			Winter					
	
Marks	July^2^	August	September	January	February	March	Mean^3^	SD^4^	Range^5^
BW1	6±0.69	11±1.3	17.3±1.8	ND	3±0.33	7±0.74	7.33±0.88	6.02	3-17.5
BW2	ND^6^	ND	ND	ND	ND	ND	ND	ND	ND
BW3	ND	ND	ND	4±0.41	4±0.39	7±0.79	2.5±0.57	2.95	2.3-7.6
BW4	ND	ND	ND	3±0.37	ND	ND	3±0.37	1.22	2.9-3.2
BW5	4±0.44	9.3	30.35	5±0.51	8±0.82	ND	4.83±0.68	3.31	3-9.6
BW6	ND	ND	5±0.55	8±0.77	8±0.79	12.2±1.3	5.5±0.89	4.81	5-12.4
BW7	3±0.32	8±0.83	ND	3±0.29	ND	ND	2.33±0.34	3.14	3-8.2
BW8	6±0.55	20.27	7±0.86	ND	7±0.7	6±0.57	4.68±0.51	2.94	1.9-7.1

1 Maximum Permissible Concentration.

2 Mean of 9 samples for each mark of bottled water.

3 Mean of 54 samples of bottled water of marks (Summer and Winter).

4 Standard Deviation.

5 Range of lead concentration for each mark.

6 Not Detected.

**Figure 1:**
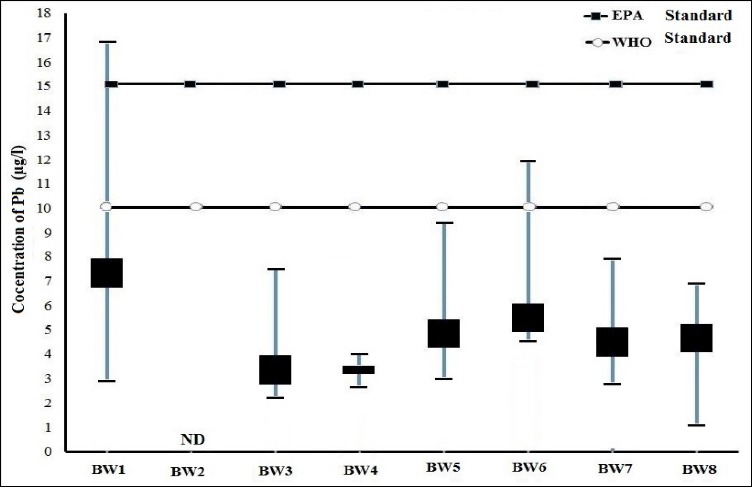
Comparison of mean and range of lead concentration in bottled water with standard of WHO and EPA

**Figure 2:**
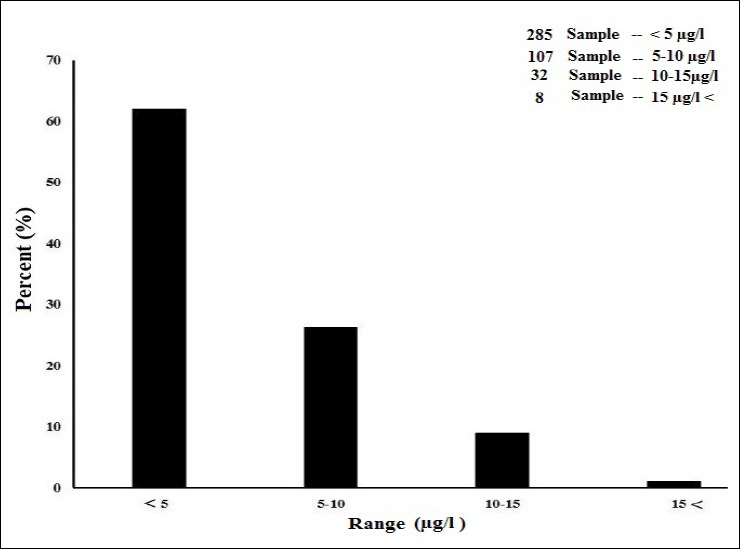
The Percentage of relative frequency distribution of lead concentration in the bottled water of Bandar Abbas (n=432)

There is a significant difference between the mean concentration of lead in the marks BW3 (p value=0.02), BW4 (p value=0.04) and BW6 (p value=0.035) and BW7 (p value= 0.03) in the summer and winter which is less than 0.05 (p value <0.05). But generally, a significant difference was not observed between the mean concentration of lead in the bottled water in summer, which was 3.38±0.41 and winter, which was 3.54±0.49 µg/l (p value <0.05). The order of lead concentration in the bottled water marks is as the following manner: BW2<BW4<BW7<BW3<BW8<BW5<BW6<BW1. The range of lead concentration for the marks BW1, BW2, BW3, BW4, BW5, BW6, BW7 and BW8 are 0-17.5, 0-0, 0-7.6, 0-3.2, 0-9.6, 0-12.6, 0-8.2, and 0-7.1 µg/l, respectively. The most and the least range are respectively, related to the marks BW1 and BW4. The most and the least percentage of frequency distribution are related to (285 Sample) <5 µg/l and (8 Sample) >15 µg/l (figure 2). 9.2 percent of samples (40 samples) have the higher concentration than the standard of WHO and 1.85 percent (8 samples) have the higher concentration than the standard of EPA.

## 4. Discussion

**Figure 3:**
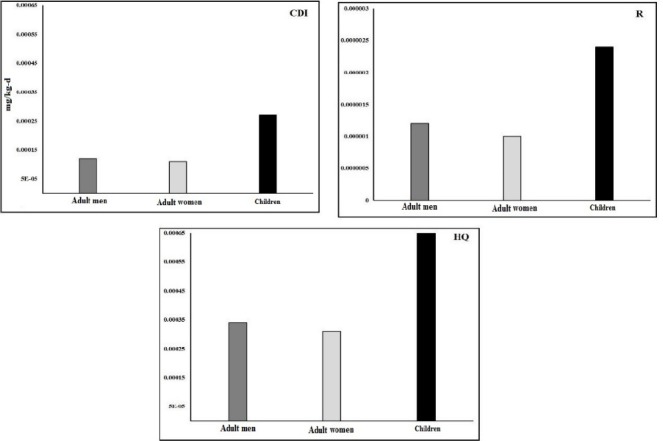
CDI, R, HQ in adult men, adult women and children due to lead in the bottled water

The mean concentration of lead in our study is 3.46±0.47 µg/l which is very close to obtained results by Ghaderpoor et al., in the field of lead concentration in the bottled water in Tehran which is 3.18±3.44 µg/l. But in contrast to our studies, the lead concentration in all the samples of GhaderPoor et al.’s study was less than the standard of WHO and EPA (Ghaderpoor, Jahed, & Nazmara, 2009). The mean concentration of lead in 15 mark of the bottled drinking water in Miranzadeh et al’s study was 10.5±0.12 µg/l which was higher than the mean in our study (Miranzadeh, Hassani, Iranshahi, Ehsanfard and Heidari, 2011). In Bakirder et al.’s study, the range of lead concentration in the bottled water is 1.78±0.1 – 4.82± 0.26 µg/l which was in the range of lead concentration in our study (1.9-17.6 µg/l) (Bakırdere, Yaroğlu, Tırık, Demiröz, & Fidan, 2013). Also, the range of lead concentration in drinking water in Ficket et al.’s study was less than the range of lead in our study (<0.01-0.042 µg/l) (Fiket, Roje, Mikac, & Kniewald, 2007). These changes of lead concentration in different areas are due to the difference in water resource (surface or groundwater), the kind of water purification process, and the existence of possible pollution (Ahmed Baig, Gul Kazi, Qadir Shah, Abbas Kandhro, & Imran Afridi, 2010). The mean CDI caused by lead for age groups of adult men, women and children is respectively 12E^-5^, 11E-5 and 27E^-5^ µg/l. The most CDI is related to BW1 mark (children age group) and the least CDI is related to the mark BW3 for adult women and BW7 for adult men and women (See Table 2). The order of mean CDI for different age groups is as following manner: children> adult women> adult men (figure CDI-3). The mean CDI for age groups of adult men in our study (12E-5) was observed more than mean CDI in Mohammad et al study (0.00007 mg/kg-d groundwater water and 0.00003 mg/kg-d surface water in the Jijal-Dubair area. In spite that the mean concentration of lead in drinking water in our study, which was 3.46 µg/l, was less than Muhammad et al.’s study, which was 3.64 µg/l, but since the DI (Daily Intake) was more for adult men (2.723 l/d), CDI in our study was also obtained more as compared (Muhammad, Shah, & Khan, 2011). There was a significant difference between the mean R of adult men and women age groups with children age group (P value <0.05). There was not a significant difference between the mean R in men and women age group (P value>0.05). The mean R of lead for children is 24E^-7^ which is about 2.4 times as much as men age group (11E-7) and women age group (10E-7) (See Figure 3-R). The more R for children age group as compared with men and women is caused by the less weight (about 60% less) and approximately equal water consumption to adult men and women age groups. The order of R is as following manner: Children> adult men>adult women. The most R is related to the BW1 mark (children, 50E^-7^) and the least one is related to the BW3mark (adult men, 7E-7), and BW7 (adult women, 7E-7). 50 percent of the marks (BW1, BW5, BW6, and BW8) for adult men and women and all the marks (100%) for children are more than the acceptable level of the R of EPA which is 1E-6. The R of all the marks is lower than the R of WHO which is 1E -4. The mean HQ for the age groups of adult men, adult women and children are respectively 34E-5, 31E-5 and 84E-5.

**Table 2:** CDI, R and HQ in adult men, adult women and children due to lead in the bottled water of Bandar Abbas

Marks	C (µg/l)	CDI (Mg/kg-d)			R			HQ		

		Adult men	Adult women	children	Adult men	Adult women	children	Adult men	Adult women	Children
BW1	7.33	26E-5	24E-5	59E-5	22E-7	21E-7	50E-7	72E-5	67E-5	164E-5
BW2	NC*	NC	NC	NC	NC	NC	NC	NC	NC	NC
BW3	2.5	9E-5	8E-5	20E-5	8E-7	7E-7	17E-7	24E-5	23E-5	56E-5
BW4	3	10E-5	10E-5	23E-5	8E-7	9E-7	19E-7	26E-5	27E-5	67E-5
BW5	4.83	17E-5	16E-5	39E-5	15E-7	14E-7	33E-7	48E-5	44E-5	108E-5
BW6	5.5	20E-5	18E-5	44E-5	17E-7	16E-7	38E-7	54E-5	50E-5	123E-5
BW7	2.33	8E-5	18E-5	19E-5	7E-7	7E-7	16E-7	23E-5	21E-5	51E-5
BW8	4.67	17E-5	16E-5	38E-5	14E-7	13E-7	32E-7	46E-5	43E-5	104E-5
Mean	3.46	12E-5	11E-5	27E-5	11E-7	10E-7	24E-7	34E-5	31E-5	84E-5

* Not Calculated.

The order of HQ in the age groups is as following manner: Children > adult men> adult women (See Figure 3 -HQ). Like R, there was a significant difference between HQ for children age group and adult men and women (p value <0.05). But there is no a significant differences between adult men and adult women (p value >0.05). The HQ for children is 2.47 times as much as adult men and women. The most HQ is related to the mark BW1 (children, 164E-5) and the least one is related to the mark BW7 (adult women, 21E-5). The HQ for all groups in the all marks of bottled water is less than 1. The R of lead in drinking water for adult age group in Muhammad et al.’s research was less than 1 (Muhammad, Shah, & Khan, 2011). The HQ in Navidollah et al’s study, which was HQ=0.664, was observed much more than it in our study (1952 times as much as in our study). Such a large difference is due to a high Pb concentration in drinking water. In Lim et al.’s study, as in our study, the R was observed less than 1 which this issue is due to the proximity of lead concentration in this study, which is 3.04-6.697 µg/l with our study (Lim, Shaharuddin, & Sam, 2012). Also, in Rajaei et al.’s study, the HQ was 0.00007 which was observed less than 1 as in our study (Rajaei, Pourkhabaz, & Hesari Motlagh, 2012). The mean concentration of lead in all the marks of the bottled water in Bandar Abbas is less than the standard of WHO and EPA. Hence, there is not a significant difference between the mean concentration of lead in the bottled water in summer and winter (p value>0.05). 82.8 percent (432 samples) of all the samples has the lead concentration lower than the standard of WHO and EPA. The R for all the age groups is more than acceptable level of EPA which is 1E-4. Children, as compared with adult men and women, are twice as likely to be more on carcinogenic and HQ (p value<0.05). The HQ for all the age groups is less than 1. At last, it can be said that the population of Bandar Abbas is in the danger area (especially for children) in terms of R due to lead in bottled drinking water (regarding acceptable levels of EPA) but they are in a safe area in terms of HQ<1.

## 5. Conclusion

The mean concentration of lead in all the marks of the bottled water in Bandar Abbas is less than the standard of WHO and EPA. Hence, there is not a significant difference between the mean concentration of lead in the bottled water in summer and winter (p value>0.05). 82.8 percent (432 samples) of all the samples has the lead concentration lower than the standard of WHO and EPA. The R for all the age groups is more than acceptable level of EPA which is 1E-4. Children, as compared with adult men and women, are twice as likely to be more on carcinogenic and HQ (p value<0.05). The HQ for all the age groups is less than 1. At last, it can be said that the population of Bandar Abbas is in the danger area (especially for children) in terms of R due to lead in bottled drinking water (regarding acceptable levels of EPA) but they are in a safe area in terms of HQ<1.
